# Supplementary material for: Curcumin Reduces Depression in Obese Patients with Type 2 Diabetes: A Randomized Controlled Trial
Source: Nutrients. 2024 Jul 25;16(15):2414. doi: 10.3390/nu16152414 (PMC11314607; doi:10.3390/nu16152414)
Supplement: Supplementary file 1 [file nutrients-16-02414-s001.zip › nutrients-3096913-supplementary.pdf]

Supplementary Materials

Supplementary Figure S1: Trial profile (CONSORT Diagram)

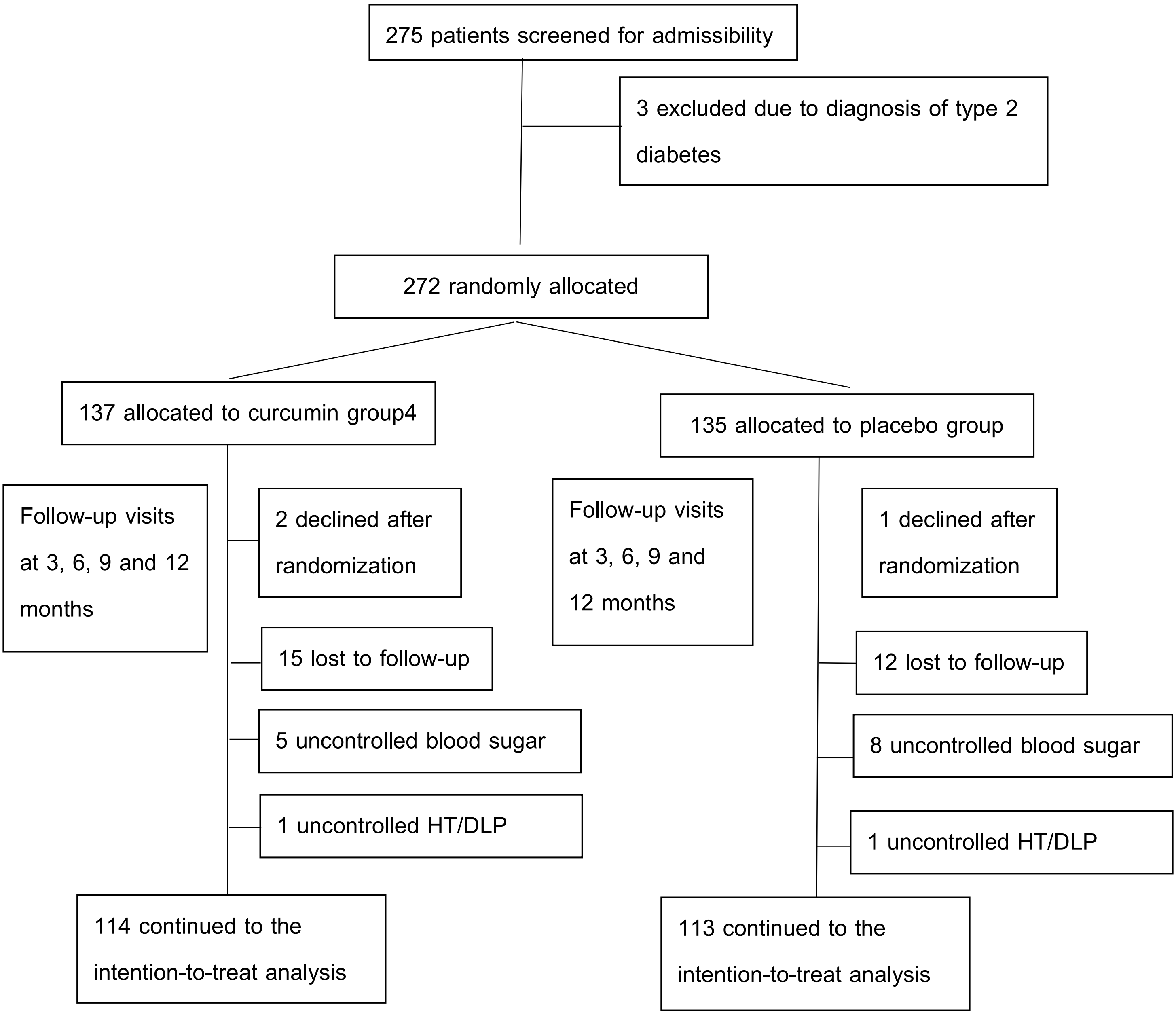

DLP, dyslipidemia; HT, hypertension

Supplementary Figure S2: Chromatographic Fingerprints of Curcuminoids Extracts

The high-performance thin-layer chromatography (HPTLC) chromatogram of the Thai Government Pharmaceutical Organization (GPO) is shown in Figure 2, compared with HPTLC chromatogram of standard across curcumin (curcuminoids) in Figure 1. In every batch of GPO curcuminoid extracts, the peak ratio of curcumin to demethoxycurcumin to bisdemethoxycurcumin was controlled to be 1 to not more than 0.6 to not more than 0.4.

Calibration: M: CURMIN
Method: Scan Integration
Analysis a:

No name
Calibration Data END

Peak	Height	Area
1	12.1	401.8
2	29.2	1385.1
3	61.1	1878.2

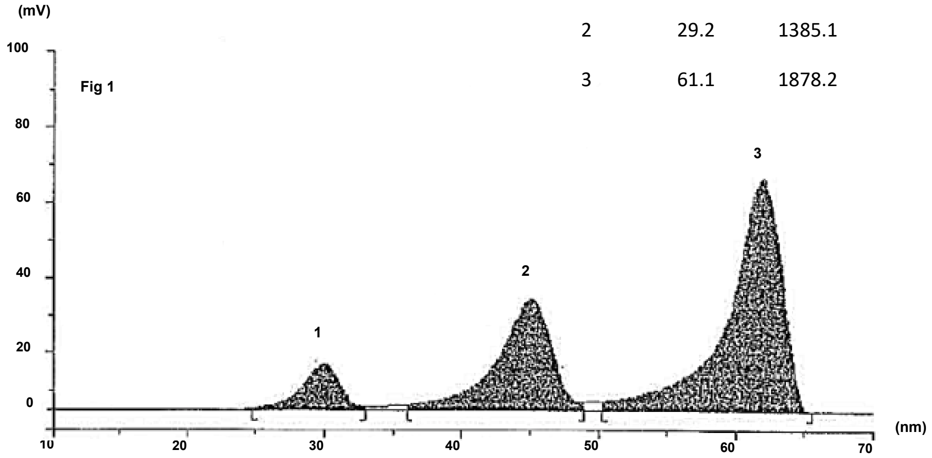

Wavelength: 420 nm

Track: 1, noise level: 0.019 mV, raw data file: 061011

Calibration: M: CURMIN
Method: Scan Integration
Analysis a:

No name
Calibration Data END

Peak	Height	Area
1	18.2	620.7
2	32.2	1528.7
3	68.5	3301.2

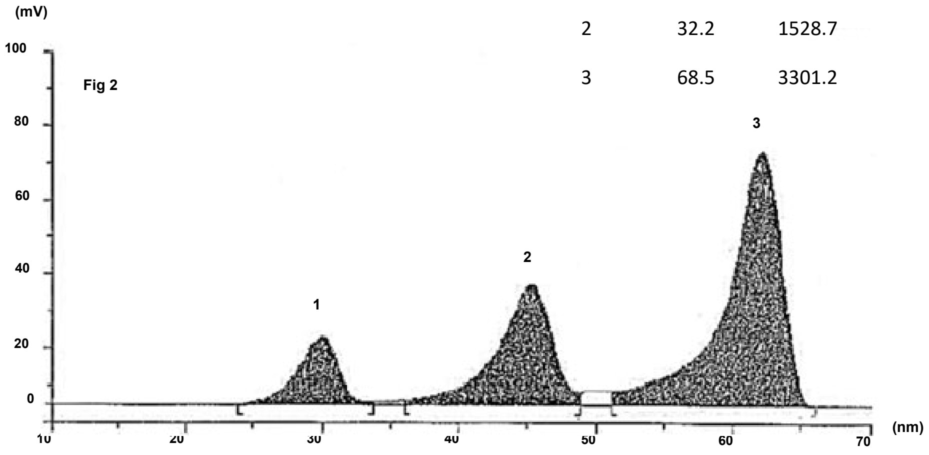

Wavelength: 420 nm

Track: 2, noise level: 0.019 mV, raw data file: 061011

Supplementary Table S1: Antihypertensive and Antidyslipidemic Medications

Medications	Placebo (n=114)	Curcumin (n=113)	p-value†
Antihypertensive medications*			
Angiotensin receptor blockers	80 (70.2)	86 (76.1)	0.39
Calcium channel blockers	26 (22.8)	18 (15.9)	0.25
Beta blockers	21 (18.4)	17 (15.0)	0.61
Antidyslipedemic medications*			
Statins	59 (51.8)	55 (48.7)	0.74

* Values expressed as number (percentage).

† Chi-square test

Supplementary Table S2: Mean Daily Intake of Nutrients at Baseline and at 12 Months, by Group

Daily Intake of Nutrients	Placebo (n=114)		Curcumin (n=113)		*P* Value[2]
	Baseline[1]	12 mo	Baseline[1]	12 mo	
Energy (kcal/d)	1857.60±110.97	1893.74±74.30	1864.21±87.98	1881.16±67.23	0.100
Carbohydrate (% of energy)	57.50±2.57	58.07±2.56	57.04±1.52	58.08±1.62	0.148
Protein (% of energy)	12.98±2.13	13.21±1.35	13.33±1.28	13.44±1.20	0.418
FAT (% of energy)	28.27±2.12	28.91±1.91	28.47±2.42	28.44±2.23	0.056
Fiber (g/d)	8.54±1.16	8.46±0.88	8.49±0.82	8.39±0.64	0.464

[1] All parameters are presented as means ± SDs. There are no significant differences between the two groups at baseline for any variable by *t* test.
[2] The curcumin had no significant effect on mean daily intake of nutrients by one-factor ANCOVA with the baseline value as the covariate.
There were no significant differences in the daily mean-energy (energy, carbohydrate, protein, fat, and fiber) and nutrient intakes between the curcumin and placebo groups.

Supplementary Table S3: Capsule Consumption by Subjects Per Day and Per 3 Months, Counted at 3-, 6-, 9-, and 12-Month Visits

	Visit	Placebo		Curcumin		*P* value
		Number of Subjects Assessed	Number of Capsules Taken *	Number of Subjects Assessed	Number of Capsules Taken *	
Consumption Per 3 Months	3 mo	114	558.69 (54.59)	113	559.13 (52.11)	0.58
	6 mo	114	558.55 (21.61)	113	556.72 (25.61)	0.13
	9 mo	114	517.16 (20.32)	113	514.96 (21.33)	0.43
	12 mo	114	515.06 (20.82)	113	514.11 (21.78)	0.33
Consumption Per Day	3 mo	114	6.21 (0.60)	113	6.21 0.58)	0.57
	6 mo	114	6.20 (0.23)	113	6.19 (0.28)	0.13
	9 mo	114	5.70 (0.22)	113	5.72 (0.24)	0.43
	9 mo	114	5.65 (0.21)	113	5.85 (0.25)	0.33

* The data are presented as the mean ± SEM

Supplementary Table S4: PHQ-9 Score, Body Composition and Chemistry Biomarker Measures Between Males and Females in Curcumin Group

Outcomes	Follow-Up Period (mo)	Males		Female		*P* Value
		**Mean**	**Minimum-Maximum**	**Mean**	**Minimum-Maximum**	
PHQ-9	0	11.64	5-15	11.55	5.00-15.00	0.87
	12	7.94	2-12	7.54	2.00-12.00	0.35
Serotonin, ng/ml	0	102.41	71.28-132.00	99.16	71.28-131.20	0.34
	12	147.51	99.87-199.87	154.92	99.87-199.87	0.21
HbA1c (%)	0	6.11	4.40-4.30	6.40	4.8-9.5	0.13
	12	5.98	4.70-7.60	6.20	4.2-8.1	0.07
Glucose (mg/dl)	0	125.22	79-175	121.92	90-178	0.30
	12	115.98	70-160	114.74	82-160	0.65
HOMA-IR	0	5.09	1.20-14.20	5.46	1.90-13.1	0.25
	12	4.60	1.20-11.00	4.94	1.60-10.20	0.35
IL-1β (pg/ml)	0	0.41	0.03-0.88	0.42	0.11-0.88	0.77
	12	0.34	0.12-0.87	0.30	0.10-0.74	0.81
IL-6, pg/ml	0	9.05	7.04-10.56	8.89	7.04-10.56	0.52
	12	6.58	3.09-10.40	5.85	3.1-12.4	0.20
TNF-α, pg/ml	0	4.77	2.64-7.04	4.71	2.64-7.04	0.88
	12	3.47	1.33-8.59	3.49	1.11-6.55	0.82
TAS, μmol trolox eq/l	0	1.61	1.25-1.87	1.56	1.35-1.86	0.09
	12	1.88	1.58-2.15	1.83	1.56-2.21	0.10
RANSEL, U/ml	0	6,433.24	1,124-13,012	6,837.51	1,822-11,969	0.25
	12	13,103.10	5,787-20,986	13,179.29	7,894.00-20,987	0.69
RANSOD, U/ml	0	245.54	144-362	231.09	163-360	0.15
	12	330.60	261-408	322.64	215-440	0.49
MDA, μmol/l	0	1.93	1.20-3.02	1.95	1.20-3.23	0.82
	12	1.42	0.93-2.60	1.47	0.99-2.54	0.57
BMI, kg/m^2	0	26.50	20.50-33.59-	27.34	20.40-36.58	0.34
	12	24.46	17.94-42.24	24.66	17.9-35.55	0.92

Supplementary Table S5: Parameters and Adverse Effects in the Curcumin-Treated and Placebo-Treated Groups at Each Follow-Up Visit

Variables	Visit	Placebo		Curcumin		*P* value
		Mean (SEM)	Min-Max	Mean (SEM)	Min-Max	
Creatinine (mg/dL)	Baseline	0.87 (0.02)	0.40-1.69	0.86 (0.02)	0.45-1.6	0.77
	3 mo	0.88(0.02)	0.45-1.81	0.91 (0.05)	0.46-7.26	0.64
	6 mo	0.94(0.02)	0.47-2.04	0.92 (0.02)	0.52-1.70	0.40
	9 mo	0.94 (0.02)	0.44-1.83	0.93 (0.02)	0.54-1.81	0.51
	12 mo	0.87(0.02)	0.40-1.69	0.85(0.02)	0.45-1.60	0.77
Aspartate aminotransferase (U/L)	Baseline	25.01 (0.87)	11-89	25.34 (0.80)	13-67	0.58
	3 mo	22.45 (0.79)	9-78	23.85(0.90)	11-111	0.076
	6 mo	23.53 (1.43)	8-214	24.12 (0.98)	10-89	0.47
	9 mo	21.78 (0.63)	11-76	24.29 (1.23)	12-114	0.88
	12 mo	25.01(0.87)	11-89	25.41(0.81)	13-67	0.54
Alanine aminotransferase (U/L)	Baseline	27.58 (1.56)	5-145	30.09 (1.50)	5-118	0.08
	3 mo	24.08 (1.1)	6-101	27.49 (1.7)	6-214	0.08
	6 mo	24.74 (1.15)	7-98	28.16 (1.64)	7-186	0.18
	9 mo	23.01 (1.14)	6-117	27.50 (1.80)	6-129	0.36
	12 mo	27.58(1.56)	5-145	30.27(1.51)	8-118	0.21
